# Low Mmp 9 and VEGF levels predict good oncologic outcome in mid and low rectal cancer patients with neoadjuvant chemoradiation

**DOI:** 10.1186/1472-6890-12-27

**Published:** 2012-12-31

**Authors:** Atilla Kurt, Fatih Yanar, Oktar Asoglu, Emre Balik, Vakur Olgac, Hasan Karanlik, Sevda Tanrikulu Kucuk, Evin Ademoglu, Gulcin Yegen, Dursun Bugra

**Affiliations:** 1 General Surgery, Sivas Cumhuriyet University, Sivas, Turkey; 2 Istanbul Faculty of Medicine, General Surgery, Istanbul University, Millet Caddesi, Sehremini, Capa, 34093, , Fatih, Istanbul, Turkey; 3 Istanbul Oncology Institute, Pathology, Istanbul University, Istanbul, Turkey; 4 Istanbul Oncology Institute, General Surgery, Istanbul University, Istanbul, Turkey; 5 Faculty of Medicine, Biochemistry, Istanbul Bilim University, Istanbul, Turkey; 6 Amerikan Hospital, General Surgery, Istanbul, Turkey

**Keywords:** Apoptotic and angiogenic markers, Prognosis, Rectal cancer

## Abstract

**Background:**

The aim of this study was to evaluate apoptotic (Bcl-2, Bax expression, caspase-3 activity, and cytochrome-c) and angiogenic (MMP-9 levels and VEGF expression) markers in operable rectal cancer patients who were treated with preoperative chemoradiotherapy (CRT) followed by total mesorectal excision (TME). Understanding these factors will facilitate the identification of potential pathological responders before treatment, leading to better local control and survival rates.

**Methods:**

Between March 2006 and March 2008, 29 patients withTNM Stage III (cT3 N+) mid or low rectal cancer were included in this study. Our sample consisted of 17 males (58.6%) and 12 females (41.4%). The median age was 60 years (range 24-88 years). Biopsy samples were taken from different portions of the tumors using flexible endoscopy before neoadjuvant CRT. Preoperatively, all patients received radiation (45-50.4 gray (Gy) in 25 cycles with concurrent 5-florouracil (5-FU) chemotherapy.

**Results:**

A complete response was observed in 7 of 29 patients (24%). Bax staining was negative in 1 of the 7 patients (14%) in the pathological complete response (PCR) group and in 18 of the 22 patients (82%) in the no pathological complete response (noPCR) group (p = 0.001). MMP-9 and VEGF levels were higher in the noPCR group than the PCR group (p = 0.04, p = 0.05 respectively). No statistically significant differences were found between VEGF and MMP-9 levels in nodal downstaging. No statistically significant relationships were found between the other apoptotic factors (Bcl 2, cytochrome-c, and caspase-3 activity) and pathological response rate (p > 0.05).

**Conclusion:**

In neoadjuvant CRT patients, high levels of Bax expression and low levels of VEGF and MMP-9 expression on preoperative biopsies indicate that the patient will potentially be a good pathological responder.

## Background

Rectal cancer (RC) is a major health problem and one of the leading causes of cancer-related death worldwide [1]. In a new multidisciplinary study of mid- and distal rectal cancer management modalities, neoadjuvant chemoradiotherapy (CRT) or radiotherapy (RT) was used for clinically staged T3/T4 and N + patients [2]. However, the primary treatment modality for patients with rectal cancer is surgery. Preoperative CRT offers a number of benefits, such as tumor volume reduction, tumor downstaging, more tumor resectability, a high rate of anal sphincter preservation, a low local recurrence rate, and improved survival [2,3]. However, a significant percentage of patients respond poorly to neoadjuvant CRT [3].

A pathologic complete response (PCR), characterized by the sterilization of all tumor cells in the resected specimen, is one of the best surrogate markers of an excellent prognosis [4-8]. Approximately 15-30% of patients will experience a PCR, whereas the majority of patients will have some degree of residual disease after CRT [4,5].

However, preoperative CRT adversely affects the functional results after surgery by decreasing the mean resting anal canal pressure and causing internal sphincter fibrosis [9], and it can also have a negative effect on sexual functioning in both genders [10]. Long-term bowel dysfunction is more prevalent in irradiated patients than in patients who underwent surgery alone [11]. In addition, rectal cancer patients treated with neoadjuvant radiotherapy have an increased risk of secondary cancer [12]. Thus, if patients with tumors that are highly responsive to CRT could be identified at the time of diagnosis, then preoperative CRT could be administered in a more selective and individualized manner.

Apoptosis is directly related to tumor pathogenesis and progression in rectal cancer. Treatment modalities such as RT may induce apoptosis in rectal tumors. Angiogenesis also plays a major role in cancer pathogenesis [13-15]. In solid tumors, angiogenesis is closely associated with the over-expression of VEGF in tumor cells [16]. Thus, evaluating apoptosis and angiogenesis will facilitate the identification of rectal cancer patients who may benefit from neoadjuvant therapy.

Several markers have been proposed as predictors of the radio- and chemosensitivity of rectal cancers, including Bcl-2, Bax, epidermal growth factor receptor (EGFR), cyclooxygenase 2 (COX 2), microsatellite instability (MIS), mismatch repair (MMR) genes, Ku-70, Ki-67, vascular endothelial growth factor (VEGF), apoptotic rate, proliferative index, and global gene expression [5,17]. To date, however, no marker reliably predicts tumor CRT response in clinical practice.

The aim of this study was to evaluate the effects of apoptotic (Bcl-2, Bax, caspase-3, and cytochrome-c expression) and angiogenic (MMP-9 levels and VEGF expression) factors on the oncological outcomes of mid- and distal rectal cancer patients managed with preoperative CRT followed by total mesorectal excision (TME). An understanding of these factors may allow potential pathological responders to be identified before treatment and may help achieve better local control and survival rates.

## Methods

### Patients

Between March 2006 and March 2008, 91 rectal cancer patients were assessed preoperatively at the Istanbul University, Istanbul Faculty of Medicine, Department of General Surgery. All data were collected and recorded prospectively, and the clinical and pathological features were reviewed retrospectively. The study was approved by the Ethics Committee of the Istanbul University Faculty of Medicine. Informed consent was obtained from all patients.

Patient treatment was planned by a multidisciplinary team, including surgeons, medical oncologists, radiation oncologists, pathologists and radiologists. Sixty-two patients were excluded for meeting one or more of the following criteria: proximal localization of the tumor (n = 22, 35%), synchronous tumors (n = 5, 8%), polyposis coli (n = 2, 3%), urgent operation (n = 4, 6%), peritonitis carcinomatosa (n = 1,%1), systemic metastasis (n = 3, 4%), surgery performed at another center (n = 2, 3%), unavailable or inadequate pretreatment archival biopsy specimens (n = 3, 4%), refusal to take part in the study (n = 15, 24%), and early stage diagnosis without preoperative CRT (n = 5, 8%). After excluding these 62 patients, 29 remaining patients were included in the study. The inclusion criteria included biopsy-proven adenocarcinoma of the mid or low rectum, clinical or radiological evidence of a T3 or T4 tumor and lymph node metastasis (cTNM, Stage III). The preoperative work-up included a general clinical examination, a digital rectal examination, a complete blood count, a blood chemistry profile, a carcinoembryonic antigen (CEA) assessment, a rigid proctosigmoidoscopy, a total colonoscopy, a tumor biopsy, computed tomography (CT) of the abdomen and a chest x-ray. Endorectal coil or pelvic phase array magnetic resonance imaging (MRI) and CT of the thorax were routinely used to i mprove preoperative staging to select patients who were candidates for preoperative CRT. Biopsy samples were taken from different parts of the tumors using flexible endoscopy. These samples were frozen and preserved at -80°C.

### Preoperative chemoradiotherapy

As is standard at our institution all patients received preoperative radiotherapy (180 cGy per day, 5 days per week in 25 fractions over a period of 5 weeks for a total of 4,500 c-Gy) with concurrent chemotherapy (5-fluorouracil 225 mg/m^2^ per day, 1 infusion for 5 days per week over a period of 5 weeks). The fields extended from the L5/S1 level superiorly to the bottom of the obturator foramina inferiorly with a 1- to 1.5-cm margin on the bony pelvic inlet. Surgical resection with curative intent was undertaken 8 weeks after the completion of CRT.

### Surgery

Twenty-nine cTNM stage III mid- or distal rectal cancer patients underwent potentially curative surgery according to the principles of TME described by Heald [18]. Sphincter-preserving surgery (SPS) was performed in 22 patients (76%), and 17 of these surgeries (77%) were performed laparoscopically. Abdominoperineal resection (APR) was performed in 7 patients (24%), and 4 (57%) of these procedures were performed laparoscopically. All patients were operated by the same surgeon (O.A) who is specialized on laparoscopy and colorectal cancer treatment. Histopathological examination of the operative specimens was performed according to the principles of Quirke et al. [19]. All the specimens were examined by the same pathologist (V.O). The pathology report included tumor staging according to the American Joint Committee on Cancer TNM staging system (6th ed.) [20].

### Laboratory methods

Biopsy samples were fixed in 10% buffered formalin for 1 week. Five-to-seven micron thick sections cut from routinely prepared paraffin blocks were stained with H&E and examined under a light microscope. Additional sections placed on positively charged slides were utilized for immunohistochemical examination. After deparaffinization, microwave antigen retrieval was performed. Thereafter, the Peroxi-Block (Ultra V block) TP 125 – HL Lab Vision Corporation Fremont, California USA process was performed, and the Primary Monoclonal Mouse Antibody to Bax® (PDM 103 clone 2D2 ready-to-use; Diagnostic Biosystems California USA) and Bcl-2® (PDM 106 clone 124; Diagnostic Biosystems California USA) were utilized. Nuclear staining was performed with hematoxylin. To determine staining density, 100 cells were counted in 3 different areas under high magnification, and based on the number of cells that were positively stained, <10% was defined as negative and >10% was defined as positive.

The MMP-9 assay was performed with a Human MMP-9 ELISA kit (Bender MedSystems GmbH, Vienna, Austria). The cytochrome-c and VEGF assays were performed with BioSource® ELISA kits (Invitrogen Corporation, CA, USA). Caspase-3 activity was measured using Apo*Target*™ colorimetric caspase-3 kit® (Invitrogen Corporation, CA, USA). P-nitroaniline, produced by the enzymatic hydrolysis of acetyl Asp-Glu-Val-Asp p-nitroaniline (Ac-DEVD-pNA), was measured using a p-nitroaniline norm at 405 nm and a molar absorptivity (10,500 M^-1^ cm^-1^) coefficient. The endoscopic tissue homogenate was centrifuged at 15,000 × *g* for 5 min at 4°C. The supernatant was added to the ELISA plate, and after incubation at 37°C for 2 hours, the absorbance was measured at 405 nm. The amount of p-nitroaniline was calculated using an extinction coefficient and a standard curve after removing the absorbance values of cells treated with caspase-3 substrate and caspase-3 inhibitor. Enzyme activity was calculated as milligrams of protein per minute according to the amount of p-aniline released. Protein assays were performed using the bicinchoninic acid method.

### Assessment of pathological responses to preoperative CRT

The response to preoperative CRT was assessed at the pretreatment clinical stage rather than the final pathologic stage. PCR was defined as the absence of any residual cancer cells in the specimen (ypT0N0). Regressive changes, including the extent of fibrosis and the amount of tumor remaining in the resected specimen, were assessed on H&E-stained slides. The pathologic response to preoperative CRT (tumor regression grade, TRG) was scored based on the grading classification described by Dworak et al. [21]: Grade 0 = no regression; Grade 1 = minimal regression, dominant tumor mass with obvious fibrosis and/or vasculopathy; Grade 2 = moderate regression, dominantly fibrotic changes with tumor cells or groups (easy to find); Grade 3 = good regression, very few (difficult to find microscopically) tumor cells in fibrotic tissue with or without mucous substance; and Grade 4 = total regression, no tumor cells, only fibrotic mass.

In this study, the group with Dworak [21] scores of 0 to 3 (partial responders or no responders, no PCR) were compared with the group with Dworak scores of 4 (complete responders, PCR).

### Statistical analysis

Differences between continuous variables were tested using either Student’s *t* test or the Wilcoxon Rank Sum Test. For categorical data, the summary statistics consisted of proportions, and comparisons were performed with either the Chi-squared Test or Fisher’s Exact Test. The cumulative probabilities of overall survival (OS) and disease-free survival (DFS) were estimated with Kaplan-Meier survival methods, and differences between subgroups were assessed with the log rank test. The duration of follow-up was calculated as the time from surgery to the event of interest. Patients without events of interest were assigned the date of the last follow-up visit. In cases of local or distant metastasis, the event was recorded and computed at its time of occurrence. To better assess the oncological implications of PCR, the Cox proportional hazards model was used to adjust the hazard ratios and corresponding 95% confidence intervals. Statistical significance was set at 5% (p < 0.05). The data were analyzed using the SPSS Program for Windows (Version 12.0; SPSS, Inc., Chicago, IL, USA).

## Results

### Patient characteristics

The sample included 29 patients (17 males [58.6%] and 12 females [41.4%]) who underwent neoadjuvant CRT followed by TME for stage cT3N + mid or low rectal cancer. The median age was 60 years (range 24-88). The distance from the anal verge was less than 5 cm in 13 patients (45%) and between 5.1 and 10 cm in 16 patients (55%). Upon gross pathological examination, the median tumor size was 30 mm (range 20-60 mm).

### Histopathological examination

Histopathological examination revealed that the differentiation of the adenocarcinoma was moderate in 25 patients (86%). Tumor regression was staged based on the scale described by Dworak: TRG 1, 5 patients (17%); TRG 2, 9 patients (31%); TRG 3, 8 patients (27%); and TRG 4, 7 patients (24%). Thus, PCR was achieved in 7 patients (24%), and noPCR was achieved in 22 patients (76%). No statistically significant difference was found between the PCR and noPCR groups in terms of age, sex, tumor distance from the anal verge, tumor localization, operation type or total number of harvested lymph nodes (p > 0.05).

### Association of apoptotic and angiogenic markers and response to CRT

Bax expression was statistically significant for T staging (p = 0.001) but was not statistically significant for N staging (p = 0.23) (Table 1). No statistically significant difference was found for Bcl-2 expression with respect to either T staging (p = 0.59) or N staging (p = 0.52) (Table 1).

**Table 1:** Relationship between Bax expression, Bcl-2 expression the expression of VEGF, Cytochrome-c, Caspase-3, or MMP-9 and T stage or N stage

	**Patients (n)**	**VEGF**	**Cy-c**	**Casp-3**	**MMP-9**	**Bax expression**	**Bcl-2 expression**
						<10%--->10%	<10%--->10%
T0	7	129.7 ± 67.7	68.3 ± 54.6	0.22 ± 0.1	488.6 ± 405.1	1(14)----6(86)	4(29)-----3(20)
T2-T3	22	516.51 ± 69.63	54.48 ± 28.85	0.07 ± 0.043	1166.6 ± 745.9	18(82)--4(18)	10(71)---12(80)
P value		0.051	0.72	0.19	0.04	0.001	0.59
						<10%--->10%	<10%--->10%
No	19	381.4 ± 631.1	61.2 ± 38.6	0.12 ± 0.2	991.4 ± 803.6	11(58)---8(42)	10(71)---9(60)
N(+)	10	528.67 ± 531.40	50.28 ± 28.15	0.06 ± 0.47	1064.97 ± 620.8	8(80)-----2(20)	4(29)-----6(40)
P value		0.20	0.68	0.21	0.50	0.23	0.52

Units: VEGF, pg/ml; Cy-c: Cytochrome-c, ng/ml; Casp-3: Caspase-3, pmol AMC/min/mg; MMP-9, ng/ml.

*Numbers in parentheses indicate percentages.

The expression of both Bax and MMP-9 differed significantly between the PCR and PPR groups: (p = 0.003) and (p = 0.04), respectively. This difference was not observed for the expression of Bcl-2, cytochrome-c, or caspase-3. The groups differed in terms of VEGF expression, but the difference was not statistically significant (p = 0.05) (Table 2). A low level of MMP-9 was significantly correlated with T downstaging seen between clinical and pathological stagings (p = 0.04). VEGF was not significant for T downstaging, (p = 0.05). There was no relationship between the expression of cytochrome-c or caspase-3 and T downstaging. None of the apoptotic or angiogenic factors showed a statistically significant association with T downstaging (Table 1).

**Table 2:** Relationship between the response to neoadjuvant CRT and the expression of Bax, Bcl-2, Cytochrome-c, Caspase-3, VEGF, and MMP-9

	**Complete response (PCR)**	**Partial or no response (noPCR)**	**p value**
Bax			
<10%	1 (14)	18 (82)	0.003
>10%	6 (86)	4 (18)	
Bcl-2			
<10%	4 (57)	10 (45)	0.59
>10%	3 (43)	12 (55)	
Cytochrome-c	68.38 ± 54.59	54.48 ± 28.85	0.4
Caspase-3	0.22 ± 0.353	0.07 ± 0.43	0.2
VEGF	129.67 ± 67.726	516.51 ± 649.63	0.05
MMP-9	488.60 ± 405.16	1166.64 ± 745.91	0.04

Units: Cytochrome-c, ng/ml; Caspase-3, pmol AMC/min/mg; VEGF, pg/ml; MMP-9, ng/ml.

*Numbers in parentheses indicate percentages**.**

### Association of apoptotic and angiogenic markers and DFS/OS

There was no postoperative mortality. During the median 50 months of follow-up, there was no local or systemic recurrence in 7 patients in the PCR group. Local or systemic recurrence was observed in 8 patients in the noPCR group (36.3%). At the end of the 50-month follow-up period, the OS was 100 % for the PCR group and 70.1% for the noPCR group. The 50-month DFS was 100% in the PCR group and 64% in the noPCR group. There was no statistically significant difference between groups according to OS (p = 0.58) and DFS (p = 0.08).

## Discussion

In locally advanced rectal cancer, preoperative chemoradiotherapy offers improved local control, reduced toxicity, and higher rates of sphincter preservation without compromising oncological outcome compared with postoperative treatment [22]. However, neoadjuvant treatment protocols may be inefficient in some patients. It is important to identify which patients will respond to neoadjuvant CRT.

Several molecular markers have been proposed as predictors of therapeutic response to CRT. However, to date, no molecular marker has been definitively proven to be predictive of response to CRT [5,23-31]. Garcia et al. [32] studied 132 patients with locally advanced rectal cancer treated with CRT followed by surgery. DNA from pretreatment tumor biopsies and control DNA from paired normal surgical specimens was screened for mutations and polymorphisms that are individually associated with failure to achieve a PCR after CRT. The authors found that the combination of a KRAS mutation with cyclin D1G870A (AA) and methylenetetrahydrofolate reductase (NAD[P]) C677T polymorphisms synergistically identify, with a high degree of accuracy, a subset of rectal cancer patients who do not develop a PCR in response to CRT. In light of previous studies, we evaluated whether apoptotic factors (Bcl-2, Bax expression, caspase-3 activity, and cytochrome-c) and angiogenic parameters (MMP-9 levels and VEGF expression) were correlated with the response to CRT and could predict OS and DFS.

Apoptosis is a morphologically distinct, gene-directed form of cell death that is characterized by cytoplasmic fragmentation and nuclear condensation, and it contributes to both physiological and pathological processes [33]. Some Bcl-2 family members induce apoptosis (Bax, Bad, Bid, and Bcl-X1), whereas others inhibit apoptosis (Bcl-2 and Bcl-X1). Cell homeostasis depends on a balance between these apoptotic and anti-apoptotic factors. Chang et al. [34] reported that Bax expression was significantly higher in a complete response group than in a partial response group (54% versus 29%, respectively; p = 0.017). In addition, CRT response was independent of other clinicopathologic parameters, including age, sex, pretreatment tumor size, distance from the anal verge, pretreatment stage (cT or cN), and type of operation. Tsamandas et al. [35] noted that in rectal adenocarcinoma, bax and bcl-2 proteins are frequently co-expressed with p53. Co-expression of bax with p53 protein is associated with poor clinical outcome, especially in cases without concomitant expression of bcl-2 [35]. Nehls et al. [36] investigated 92 rectal cancer patients treated with preoperative radiotherapy and found that Bax protein expression may help to predict disease recurrence in preoperatively irradiated rectal cancers, whereas expression of p53, the proposed upstream regulator of bax-induced apoptosis, did not provide additional prognostic information. In our study, bax expression was found in 86% of patients in the complete response group and in 18% of patients in the partial response group. This difference is statistically significant (p = 0.001). The results of our study, together with those of previous studies, suggest that Bax expression is important for determining the response to CRT.

The biochemical detection of caspase-3 activity is a simple quantitative method for measuring a marker of apoptosis in tissue samples and preoperative rectal cancer biopsies. The pro-apoptotic enzyme caspase-3 acts at a convergence point of the intrinsic and extrinsic apoptosis induction pathways; therefore, its activity should yield a reliable measure of ongoing levels of apoptosis in tumor samples [37]. Heer et al. [38] determined the feasibility of measuring caspase-3 activity using 47 archived fresh-frozen preoperative biopsy samples and corresponding resected rectal tumor specimens. Caspase-3 activity levels were strongly correlated in preoperative biopsies and tumor samples. The levels were significantly higher in the tumor specimens than in the preoperative biopsies. Heer et al. concluded that caspase-3 activity is an important indicator of local recurrence in rectal cancer. However, in our study, caspase-3 levels did not differ between the PCR and noPCR groups.

In the present study, we investigated another apoptotic marker, Bcl-2 expression. The role of Bcl-2 expression remains controversial. Contu et al. [39] did not find any correlation between Bcl-2 expression and age, histological grade, depth of invasion, lymphatic involvement, distant metastasis or tumor stage. Similarly, in our study, we found no significant relationship between Bcl-2 expression and pathologic response rate.

Matrix metalloproteinase expression and VEGF expression are the most investigated indicators in terms of the role of angiogenesis in the pathological response. MMP-9 is a substantial matrix metalloproteinase that plays an important role in metastasis and cancer invasion, leading to the degradation of matrix components [40,41]. In patients with colorectal cancer, MMP-9 expression in tumor tissue was found to be higher than that in healthy mucosa. Unsal et al. [42] reported that MMP-9 expression in patients with rectal cancer was correlated with poor tumor response to preoperative CRT in their study of 44 cases. In our study, the mean MMP-9 level was 488.60 ± 405.169 ng/ml in complete responders and 1166 ± 745.912 ng/ml in partial responders. This difference was statistically significant (p = 0.04).

Various regulatory angiogenic factors, such as VEGF, acidic or basic fibroblast growth factor and interleukin-8 (IL-8), work together to organize the complex process of angiogenesis. Angiogenesis is related to VEGF over-expression in tumor cells, especially in solid tumors [43].

Although VEGF was not detected in normal rectal mucosa in recent immunohistochemical studies, significant immunoreactivity was noted in rectal cancer tissues [43]. Wong et al. [44] studied the relationship between VEGF expression and progression from adenoma to carcinoma, and they emphasized that VEGF activation is an early occurrence that may promote the initiation of angiogenesis. Nozue et al. [45] investigated VEGF expression in patients with locally advanced rectal cancer before and after neoadjuvant RT and detected intense VEGF immunoreactivity and numerous VEGF-positive tumors. In the study of Zlobec et al. [46], a strong association was found between complete tumor response and the absence (or low levels) of VEGF expression in biopsy samples of rectal tumors prior to RT. VEGF expression was elevated and had notable immunoreactivity in non-responder tumors compared with complete responder tumors. In our study, the mean VEGF level was 516.51 ± 649.634 pg/ml in the partial responder group and 129.67 ± 67.726 pg/ml in the complete responder group (p = 0.05). In other words, our results suggest there may be an increase in VEGF levels in the noPCR group, similar to the findings of Zlobec et al [46].

In the present study, we also evaluated the oncological outcomes of patients with stage III (cT3 N+) low or mid rectal cancer. Pathological examination of the surgical specimens revealed PCR in 7 patients (24.1%) and noPCR in 22 patients (75.8%). Our prospective survival analysis indicates that patients in the PCR group experienced better oncological outcomes than patients in the noPCR group. In the PCR patients, the 50-month OS and DFS were both 100%. In the noPCR group, the 50-month OS and DFS were 70.1% and 64%, respectively. This difference is notable but statistically not significant (OS, p = 0.58; DFS, p = 0.08).

Our results suggest that oncological and functional outcomes after preoperative CRT are likely to be worse in patients with low bax expression and high levels of VEGF and MMP-9 upon histopathological examination of endoscopic biopsy tissues. Survival rates are better in PCR, on the other hand, low VEGF, MMP-9 levels and high Bax expression is found in patients with PCR. As a result of these findings, These parameters may be useful in prediction of PCR and different treatment options may be considered with the hope of increasing response rates in this group of patients. New chemotherapy protocols may be administered to increase the likelihood of a complete response in this group.

## Conclusions

In conclusion, our data confirm the existing evidence that rectal cancer patients have a favorable long-term outcome after preoperative CRT with a low risk of local recurrence and distant metastasis. An analysis of these markers may be useful for predicting the response rates to neoadjuvant CRT. Prospective randomized studies with large samples are needed to confirm these oncological markers for predicting the response to CRT and to design tailored neoadjuvant and adjuvant therapies on an individualized patient basis.

## Competing interests

Oktar ASOGLU, MD, Professor and other co-authors declare no competing interests.

## Authors’ contributions

OA designed the study protocol. OA carried out the preoperative assessments of the patients. OA performed the all operations, coordinated the entire study, performed the endoscopic procedures, evaluated data, designed the study protocol. OA, DB, AK, FY, EB evaluated data. VO performed the pathologic examinations. OA carried out the follow-ups of the patients, HK performed the statistical analysis. STK and EA performed the biochemical evaluations. GY assessed the pathological responses to preoperative CRT. AK, OA drafted the manuscript and DB reviewed the results. All authors read and approved the final manuscript.

## Authors’ information

Atilla Kurt was clinical fellow in General Surgery Department of Istanbul Medical Faculty during the study.

## Pre-publication history

The pre-publication history for this paper can be accessed here:

http://www.biomedcentral.com/1472-6890/12/27/prepub
